# Call to Action: Supporting Latin American Early Career Researchers on the Quest for Sustainable Development in the Region

**DOI:** 10.3389/frma.2021.657120

**Published:** 2021-05-14

**Authors:** Sandra Lopez-Verges, Fernando Valiente-Echeverría, Alex Godoy-Faúndez, David Fernandez Rivas, Bernardo Urbani, Juan José Berger, Paulina Carmona-Mora

**Affiliations:** ^1^Department of Research in Virology and Biotechnology, Gorgas Memorial Institute of Health Studies, Panama City, Panama; ^2^Virology Program, Faculty of Medicine, Institute of Biomedical Sciences, Universidad de Chile, Santiago, Chile; ^3^CiSGER, Facultad de Ingeniería, Universidad del Desarrollo, Santiago, Chile; ^4^Mesoscale Chemical Systems, University of Twente, Enschede, Netherlands; ^5^Center for Anthropology, Instituto Venezolano de Investigaciones Científicas, Caracas, Venezuela; ^6^Facultad de Economía y Negocios, Universidad Andrés Bello, President of National Association of Postgraduate Researchers of Chile, Santiago, Chile; ^7^Department of Neurology and MIND Institute, University of California Davis, Sacramento, CA, United States

**Keywords:** Early Career Researcher (ECR), science diplomacy, Latin America, sustainable development, career, scientific diaspora, sustainable development goals

## Introduction

Science diplomacy could be broadly defined as scientific interactions to tackle common concerns (science in diplomacy). These collaborations could result in positive interaction between countries (science for diplomacy) or use diplomatic interactions to increase scientific knowledge and collaboration between countries (diplomacy for science). Besides their diversity, Latin America and the Caribbean (LAC) countries have some general features that could facilitate the use of science diplomacy to strengthen their interactions for the technological development of the region (FECYT, 2017; Gual Soler, 2020). For that, each component of the scientific system in the region needs to be analyzed, creating the basis to suggest recommendations as part of the regional science diplomacy and science policy strategies. Early and mid-career researchers are crucial in the scientific system, and they create the future scientific capacity of the region. Thus, the design of science diplomacy and science-strengthening policies is critical to inform national and regional policymakers with unified and customized recommendations to improve the systems that host these ECRs.

ECRs are broadly defined as researchers under 35 years old who obtained their highest degree within the last 5–10 years and or do not yet have a permanent position (Bazeley, 2003). In the LAC context, we expand this definition (ECRs-LAC) to up to 10 years post PhD and younger than 45 years old, because compared to other regions, careers of ECRs-LAC begin later (Kreimer and Vessuri, 2018; Education at a Glance, 2021) due to older age at the beginning of the doctorate, delayed graduation rates, educational structures, and differences in opportunities. These factors impact the international competitiveness of ECRs-LAC and the scientific attractivity of the region. ECRs-LAC issues are a direct concern to researchers and institutions, and to the development strategies of LAC countries. A supportive system that enables a sustainable research career provides an important scaffold for knowledge and technological development in local or regional contexts. LAC countries have diverse levels of scientific development; but overall, the percentage of GDP allocated to research, technology, and innovation is <1% (up to 10 times lower than most high-income countries) and has been decreasing in recent years, leaving research systems in a non-competitive position (IDB, 2010; RICYT, 2019; Bolaños-Villegas et al., 2020). Less opportunities for scientific education, training, and academic positions are proposed to contribute to the high mobility of doctoral students and ECRs out of the region (Lemarchand, 2015). ECRs-LAC can be split into three mobility groups based on their professional trajectories: (1) those who pursued their professional development in their home country, (2) those who undertook part of their training abroad and then returned to their home country, and (3) those who left their home country to pursue a career and remained abroad (scientific diaspora) (Pinto-Baleisan and Delage, 2017). These career paths could inherently influence access to opportunities. Are LAC scientific systems able to compete in current knowledge production dynamics and respond to the motivations of ECRs-LAC mobility?

Some LAC countries (i.e., Brazil, Mexico, Chile, and Argentina) have their own renowned doctoral programs, allowing many ECRs to pursue their professional development at home (mobility group 1) (Lemarchand, 2015) and attracting PhD students from other LAC countries. In recent decades, many LAC governments have invested in fellowship programs that allow ECRs to undergo specialized training outside the region^1^ (IESALC, 2019). This has created unprecedented academic exchange and mobility. Such programs have had a significant impact in countries without scientific doctoral programs; but without parallel local investment, newly trained ECRs (mobility group 2) return to scientific systems that lack sufficient infrastructure and funding agencies to support their reinsertion and fully harness their training^2^ (Ramírez, 2018) (Table 1). The factors influencing ECRs-LAC mobility have not been fully harnessed to inform policies that better support their career trajectories for personal, national, and regional benefits (Dalton, 2008). Some efforts have addressed the effect of internationalization of LAC scholars through reinsertion programs that facilitate employment upon returning home^3^ Civic organizations have contributed by presenting evidence and proposed new policies impacting ECRs-LAC^4^. Independent and governmental agency-supported networks of LAC researchers create additional mechanisms of communication between researchers in the region and the diaspora^5^ (Gaillard and Gaillard, 2014) (Table 1). At the regional and ground levels, ECR organizations like the Global Young Academy (GYA), The World Academy of Sciences Young Affiliates Network (TWAS-TYAN), and National Young Academies (NYAs) continue to create new opportunities in the field of science diplomacy for LAC countries.

**Table 1:** Towards a better understanding of the challenges ECRs-LAC face to support the development of stronger scientific systems in LAC.

**Challenges/questions**	Actors^*^	**Ways forward/recommendations**
Current circumstances faced per professional mobility groups	Governments, scientific societies, and ECR organizations	Studies to understand the current circumstances of ECRs-LAC and strategic plan to equal opportunity for mobility
Access and availability of higher education opportunities	Governments, scientific societies, and ECR organizations	Increase national graduate programs and create new regional graduate programs and fellowships
Repatriation/reinsertion	Governments, scientific societies, and industry or start-up incubators/stimuli	Programs that consider not only funding but also infrastructure and institutional support
Connection with the diaspora	Governments, research institutions, scientific societies, industry or start-up incubators, diaspora organizations, and ECR organizations	Science diplomacy programs to increase collaborations and exchanges taking advantage of strengths of the science from the new country of the diaspora. Agreements and funding for partnerships. Facilitate access to research facilities
Assessing the quality of ECRs-LAC research	Governments, scientific societies, and ECR organizations	Determine what the quality of research is and its scientific or societal impacts and create better research assessment to generate a more holistic view of research performance that benefits all research fields
Impact and internationalization of ECRs-LAC research	Governments, scientific societies, research institutions, ECR organizations, and international organizations	Design specific strategies for the region on open access of research (for LAC researchers to have access to international production and for LAC publications to be read globally) publication cost, international collaboration in a win-win design (no “colonial science”)
Quantification of the public policy of research in the region	ONU, CEPAL, research institutions, and Ministries of Science or relevant divisions	Assess current policies and their actual impact/value in the research systems through a science policy lens to modify existing research-related policies or create new ones accordingly

*Here, the main challenges/questions of ECRs-LAC, along with the diverse actors that should be involved in addressing such challenges, and ways forward/recommendations to have a supportive research environment for ECRs are presented*.

* *Examples of ECRs and diaspora organizations and networks: Global Young Academy (international ECR organization), TYAN-TWAS (international ECR network from an international scientific academy), Redes Chilenas-Chile (independent ECR network including diaspora), RAICES-Argentina (governmental agency program for diaspora), RedGlobal MX-Mexico (independent diaspora network established with governmental support)*.

Through this opinion, we encourage reflection and dialogue on the issues that the ECRs-LAC face. By considering these challenges and actively participating in studies about ECRs, we hope to create strategies to better support the next generation of science change-makers in the region. The success of this study requires collaboration between ECR organizations and policymakers. Harnessing the human capital that ECRs-LAC represent is crucial for the region to meet the United Nations (UN) 2030 sustainable development goals^6^.

## Challenges Affecting ECRs, How the Situation in LAC Is Different

Globally, ECRs represent a more vulnerable group in the field of research, facing specific challenges, which may also vary between regions. The overall increase in doctorate graduates and deficient creation of new professional opportunities are resulting in increased ECR job insecurity, jeopardizing the continuity of ECRs in academia or allied industries (Editorial, 2016; Interview, 2019). These issues have been exacerbated worldwide by the COVID-19 pandemic, leading to more professional precarity, less funding, and increased job insecurity (Byrom, 2020; Editorial, 2020; Paula, 2020). The effect could be stronger in LAC, a region with lower investment in research^7^ (Bolaños-Villegas et al., 2020; Pérez Ortega and Wessel, 2020). Regional ECR-focused studies conducted by the GYA in Brazil, ASEAN (Association of Southeast Asian Nations), and Africa (Beaudry, 2014; Geffers et al., 2017; Neumann, 2018) have highlighted specific regional challenges. Mobility and career internationalization during ECR training are common. However, these are increased in Africa and LAC regions (Beaudry, 2014; Geffers et al., 2017; Neumann, 2018; Rivero et al., 2020) where international mobility is a necessity due to a lack of appropriate graduate programs or topic-specific expertise in the home country of an ECR (Castillo Jaén, 2005; Lemarchand, 2015). A better understanding of the mobility of ECRs-LAC could help design through diplomacy for science, regional strategies that support improvement in graduate programs, and research careers in the region (Table 1). This will enable ECRs-LAC to have increased opportunities to access quality training, the underlying premise creating the Instituto de Educación Superior de América Latina y el Caribe (IESALC-UNESCO).

The ECR trajectories defined earlier could also impact access to further opportunities. ECRs-LAC hired as postdoctoral fellows usually confront disadvantages based on financial constraints, lack of institutional support and retirement savings, and low salaries compared to young scientists in similar positions in developed countries (Righini and Mota, 2018). Further, the LAC private sector does not report R&D expenditures (Islam, 2014) to create opportunities for this workforce.

While many challenges of ECRs are global, research “ecosystems” (higher education and research institutions, government agencies, private sector, and relevant policies) in LAC contribute to the isolation of issues that stem, for example, from international mobility. A trend in some LAC countries to assign more value to professionals who have international training may cause bias in job prospects, hiring processes, salaries, performance evaluation (preventing objective assessment of research quality), and funding adjudication (Cantini et al., 2019; Chiappa and Perez Mejias, 2019). In a region with great social and economic inequalities, with inequitable opportunities for higher education, this bias for internationalization could perpetuate or strengthen the advantages of higher social classes (Perez Mejias et al., 2018). Accordingly, some programs may consider merits and the socio-economic level of students but more data are needed to understand the impact of such solutions^8^ (UN, Department of Economic and Social Affairs, 2020). While global experience is indeed an added value with inherent validity in terms of competitiveness and excellence, vigilance to practices in processes related to human capital management is advised. An *a priori* and subjective undervaluation of domestic education and training creates a vicious circle that threatens the quality of the same systems that are the focus of improvement. Also, an ultra-protective system benefiting national graduates, regardless of international competitiveness, is also a dangerous trend. Moreover, reinsertion of ECRs who graduated abroad into their national systems as independent researchers can be complicated by bureaucratic and time-consuming recognition systems of studies abroad^9^. This threatens international or regional agreements that aim to increase international exchange and collaboration, a relevant situation considering that LAC is one of the regions with the poorest intra-regional mobility, with countries turning to the Global North^10^. Both unbalanced internationalism and national inbreeding can be detrimental to conducive research systems. A structured assessment system for the quality of research produced by ECRs-LAC could be designed and implemented to generate a more holistic view of research performance and its impact (Table 1).

Motivations for home country return are broad and hard to assess as isolated entities. These include scientific trends, national funding guidelines, personal circumstances, instability in host countries, or a combination of many. They have been analyzed in some LAC countries (Rivero, 2018; Rivero and Peña, 2020; Stehli, 2020). Often, the main motivation emanates from funding agreements to pursue training abroad that make return mandatory. Additional programs to support repatriation and insertion of highly skilled workers through funds for research and salary^11^ (Arce Miyaki and Gomis Hernández, 2019) are key to fully harness the training pursued (Table 1). Unfortunately, oftentimes, ECRs-LAC do not have the equipment or infrastructure necessary for their research or their home institution does not hire them once a grant is completed^12^ (Barañao, 2016). Consequently, a fourth mobility subgroup is created by researchers who returned home but, because of sociopolitical or economic reasons or lack of opportunities, decided to emigrate again.

## How Regional Strategies Could Increase Competitive Research in the LAC Region

Irrespective of location, the scientific diaspora can actively contribute to knowledge development and exchange with their home country (Barré et al., 2003; Palacios-Callender and Roberts, 2018; Labrianidis et al., 2019) as their potential in science diplomacy and bilateral facilitation is a well-established notion (Burns, 2013; Wren, 2014). A well-connected diaspora may aid reinsertion strategies (Stehli, 2020) and help in designing national and regional graduate programs that could increase intraregional mobility, strengthening regional collaboration, and increasing productivity and visibility of research from ECRs-LAC (Table 1). ECRs-LAC could also pose as great science ambassadors for their countries, harnessing international connections and intermixing them in their home countries. LAC countries can actively integrate the diaspora in their science diplomacy strategies, create and strengthen scientific diaspora networks, and learn about successful cases from other countries (Gual Soler, 2020) (Table 1). Such concepts are already part of the science diplomacy approach of Spain (Elorza Moreno et al., 2017). In Latin America, successful examples of diaspora networks exist either as part of a ministerial framework (e.g., Argentina, Mexico) or as groups of independent networks (e.g., Chile)^13^. Similarly, ECRs-LAC, regardless of location, could play a role in science diplomacy and sustainable development of their country and region through government institutions and international and ECR organizations like the GYA and TWAS-TYAN. They can give a diverse perspective on ECR issues. Cross-disciplinary studies that focus on surveying the current landscape of ECRs-LAC are still needed to understand how regional scientific systems are supporting their careers (Table 1). Comparing with other regions can help discriminate general issues from specific regional ones and learn from best practices. Sub-regional associations built their research agendas based on common institutional guidelines that likely differ within LAC, originating disparities in reaching pan-regional goals^14^. The call to action is to identify the best strategies to solve roadblocks in the way of ECRs-LAC, so the region can benefit from their knowledge production. For each main challenge faced by ECRs-LAC, we suggest which essential actors should participate in the discussion to generate recommendations and ways forward to respond to these issues using data already generated or that need to be generated from multidisciplinary regional and national studies (Table 1).

## Opening the Discussion on ECRs-LAC

Identification of ECRs-LAC concerns can be instrumental in the development of supportive policies for national scientific agendas. ECR networks and international organizations that include ECRs-LAC living in the region and the diaspora should work together in designing studies to understand their particular challenges and to communicate them to relevant national and regional institutions. The diaspora can directly contribute to locally based ECRs on scientific collaboration and science diplomacy strategies that have a direct impact on the scientific progress in their home country.

While there have been efforts made to assess the status of ECRs-LAC, such as focusing on specific countries, disciplines, and aspects of their careers, a more holistic and systematic assessment is required. The GYA, in collaboration with other scientific academies like TWAS-TYAN, is undertaking this task as an ECR organization that is able to provide a voice to the diverse young researchers in the region. The study targets countries with different research profiles as a proxy of diverse LAC systems. Such evaluation in a regional and integrative approach will enable a combination of science diplomacy strategies and policies for a harmonized advancement of research in the region that could allow science-based sustainable development. This opinion article is based on currently available information on the topic and is an invitation to dialogue about ECRs in general and ECRs-LAC.

## Author Contributions

All authors contributed to the bibliographical search, discussion, writing, and editing of the article.

## Conflict of Interest

The authors declare that the research was conducted in the absence of any commercial or financial relationships that could be construed as a potential conflict of interest.

## Footnotes

^1^Examples of mobility programs in LAC that are part of a governmental scheme: https://www.senacyt.gob.pa/becas-internacionales-e-insercion-de-becarios/ http://www.dipres.gob.cl/597/articles-163122_r_ejecutivo_institucional.pdf https://minciencias.gov.co/convocatorias/formacion-y-vinculacion-capital-humano-alto-nivel/convocatoria-doctorados-en-el https://www.conacyt.gob.mx/index.php/becas-y-posgrados/becas-en-el-extranjero/estancias-posdoctorales-y-sabaticas-en-el-extranjero http://www.pronabec.gob.pe/modPublicaciones/2020/Memoria%20Anual%202018%20-%20Pronabec.pdf.

^2^Programa Inserción de becarios SENACYT-Panama https://www.senacyt.gob.pa/wp-content/uploads/2017/02/PIB-2015-Reglamento.pdf

^3^For example, the RAICES program (Argentina): https://web.archive.org/web/20110902184504/http://www.raices.mincyt.gov.ar/documentos/Programa%20Raices%202011.pdf. In Chile https://www.conicyt.cl/pai/category/lineas-del-programa/atraccion-de-cientificos-en-el-extranjero/ https://www.conicyt.cl/pai/category/lineas-del-programa/insercion-en-la-academia/.

^4^https://globalyoungacademy.net/activities/the-global-state-of-young-scientists/, https://redeschilenas.cl/2021/01/03/dic-2020-boletin-rech/v. https://anip.cl/uncategorized/el-decalogo-de-la-investigacion/?_thumbnail_id=495 https://anip.cl/insercion-laboral-de-posgraduados/ https://www.csic.edu.uy/sites/csic/files/documentos/el_apoyo_a_la_repatriacion_de_cientificos_desde_la_universidad_de_la_republica_oriental_del_uruguay_%28 2%29.pdf.

^5^Examples of LAC diaspora networks: https://redeschilenas.cl/redes-miembro-rech/ https://cancilleria.gob.ar/es/actualidad/boletin/redes-de-cientificos-argentinos-por-el-mundo https://www.cientificos.pe/ http://redtalentos.gob.mx/index.php

^6^Innovation and technology fuel economies, diverse metrics point toward investing in science for economic development: https://publications.iadb.org/publications/spanish/document/Ciencia-tecnolog%C3%ADa-e-innovaci%C3%B3n-en-Am%C3%A9rica-Latina-y-el-Caribe-Un-compendio-estad%C3%ADstico-de-indicadores.pdf.

^7^https://www.cgap.org/research/data/2018-funding-latin-america-and-caribbean https://www.cepal.org/es/comunicados/america-latina-caribe-rezagada-investigacion-desarrollo https://www.imf.org/en/News/Articles/2020/08/12/na081320-lack-of-human-capital-is-holding-back-latin-americas-growth

^8^Conversations on academic merit: https://www.acusafrica.com/post/cuppaconversation-the-limits-of-academic-merit-in-chile; Scholarships for developing countries: https://www.topuniversities.com/student-info/scholarships/international-scholarships-students-developing-countries

^9^Future analyses could show the impact of the new Regional Convention On The Recognition Of Studies, Degrees And Diplomas In Higher Education In LAC: http://www.iesalc.unesco.org/2019/07/15/23-countries-adopted-the-new-regional-convention-on-the-recognition-of-studies-degrees-and-diplomas-in-higher-education-in-lac/ http://portal.unesco.org/es/ev.php-URL_ID=49523&URL_DO=DO_TOPIC&URL_SECTION=201.html.

^10^https://monitor.icef.com/2019/10/new-scheme-aims-to-boost-student-mobility-within-latin-america/.

^11^For example, Programa Inserción de becarios, SENACYT, Panama https://www.senacyt.gob.pa/wp-content/uploads/2017/02/PIB-2015-Reglamento.pdf Programa Atracción e Inserción de Capital Humano Avanzado, CONICYT, Chile https://www.conicyt.cl/pai/sobre-pai/que-es-pai/.

^12^Pew Latin American Fellows, an example of a non-governmental program including repatriation funds: https://www.pewtrusts.org/en/research-and-analysis/fact-sheets/2014/10/a-ripple-effect-on-latin-american-science.

^13^Networks of Argentinean researchers abroad are institutionalized under the Ministry of Science, Technology and Innovation, and are intended to function as science diplomacy and policy actors: https://www.argentina.gob.ar/sites/default/files/if-2020-66820401-apn-spypctei-mct_anexo_iv_reglamento_del_sistema_de_redes_de_cientifico-as_argentino-as_en_el_exterior.pdf. An independent Mexican network was established with support of governmental agencies from Mexico https://www.redglobalmx.eu/. Redes Chilenas groups Chilean diaspora networks - mostly ECRs. Despite not being under an institutional framework, they have an impact on science policy https://redeschilenas.cl/.

^14^Subregional associations in LAC: Mercosur https://www.recyt.mercosur.int/ and CARICOM https://caricom.org/category/science-and-technology/.
